# CHOICE TO PROVIDE CARE AND KNOWLEDGE OF DEMENTIA SYMPTOMS: IMPACT ON CAREGIVERS’ EXPERIENCES

**DOI:** 10.1093/geroni/igad104.3223

**Published:** 2023-12-21

**Authors:** Sumiyyah Zimami, Hala Darwish, Deanna J Marriott

**Affiliations:** University of Michigan, Ann Arbor, Michigan, United States; University of Michigan, Ann Arbor, Michigan, United States; University of Michigan School of Nursing, Ann Arbor, Michigan, United States

## Abstract

As the population ages, understanding the needs of people with dementia and their caregivers becomes increasingly crucial. Caregivers have various experiences related to the rewards and challenges of choosing to provide care. This project investigates how caregivers’ choice to provide care for someone with dementia and their knowledge of dementia symptoms influence their caregiving experiences. The study employed a secondary data analysis from the National Alliance for Caregiving and the American Association of Retired Persons. Caregiving experiences were gauged using a 5-point Likert scale ranging from ’completely negative’ to ’completely positive’. Statistical analyses were conducted using descriptive statistics, chi-square tests, and ordinal logistic regression adjusting for covariates such as dementia stage, caregiver relationship, mental health, and caregiving attitude. Relative risks and 95% confidence intervals were estimated for interpretation using Stata/SE 18.0 with an a-priori significance level of p< 0.05. The study involves 927 caregivers, predominantly women (66.13%) aged 50 – 74. Caregivers who chose to provide care and recognized dementia symptoms were significantly more likely to report positive experiences (ARR= 1.38, p=< 0.001; ARR= 2.27, p=0.03) respectively. Caregivers who felt fulfilled or optimistic had positive experiences (ARR= 1.92, p< 0.001; ARR= 2.21, p< 0.001). Contrastingly, caregivers dealing with emotional stress or caring for relatives with moderate dementia were more likely to have a negative experience (ARR= 2.80, p< 0.001; ARR= 1.39, p=0.006). These findings highlight the importance of developing tailored nursing interventions to support caregivers’ conscious decision to provide care and increase caregivers’ knowledge, potentially enhancing the quality of life for caregivers.

